# An evaluation of minimal cellular functions to sustain a bacterial cell

**DOI:** 10.1186/1752-0509-3-111

**Published:** 2009-11-28

**Authors:** Yusuke Azuma, Motonori Ota

**Affiliations:** 1 Graduate School of Bioscience and Biotechnology, Tokyo Institute of Technology, Nagatsuta-cho, Midori-ku, Yokohama 226-8501, Japan; 2 Graduate School of Information Science, Nagoya University, Furo-cho, Chikusa-ku, Nagoya 464-8601, Japan

## Abstract

**Background:**

Both computational and experimental approaches have been used to determine the minimal gene set required to sustain a bacterial cell. Such studies have provided clues to the minimal cellular-function set needed for life. We evaluate a minimal cellular-function set directly, instead of a geneset.

**Results:**

We estimated the essentialities of KEGG pathway maps as the entities of cellular functions, based on comparative genomics and metabolic network analyses. The former examined the evolutionary conservation of each pathway map by homology searches, and detected "conserved pathway maps". The latter identified "organism-specific pathway maps" that supply compounds required for the conserved pathway maps. We defined both pathway maps as "autonomous pathway maps". Among the set of autonomous pathway maps, the one that could synthesize all of the biomass components (the essential constituents for the cellular component of Escherichia coli/Bacillus subtilis), and that was composed of a minimal number of pathway maps, was determined for each of E. coli and B. subtilis, as "minimal pathway maps". We consider that they correspond to a minimal cellular-function set. The network of minimal pathway maps, composed of 20 conserved pathway maps and 21 organism-specific pathway maps for E. coli, starts a sequence of catabolic processes from carbohydrate metabolism. The catabolized compounds are used for anabolism, thus creating materials for cell components and for genetic information processing.

**Conclusion:**

Our analyses of these pathway maps revealed that those functioning in "genetic information processing" are likely to be conserved, but those for catabolism are not, reflecting an evolutionary aspect of cellular functions. Minimal pathway maps were compared with a systematic gene knockout experiment, other computational results and parasitic genomes, and showed qualitative agreement, with some reasonable exceptions due to the experimental conditions or differences of computational methods. Our method provides an alternative way to explore the minimal cellular function set.

## Background

Advances in sequencing technology have allowed the complete genome sequences of more than 750 prokaryotes and 20 eukaryotes to be determined thus far. One of the possible subjects to be solved using this advance of data is the identification of a minimal gene set i.e. an estimation of the genes that are necessary and sufficient for sustaining a functional cell under certain conditions [1]. This type of research has attracted a lot of attention, not only for its scientific meaning, but also for its industrial applications. Both computational and experimental approaches have been employed to estimate minimal gene sets.

In the computational approach, it is assumed that the genes shared by distantly related organisms are likely to be essential, and that a catalogue of these genes might comprise a minimal gene set for cellular life [1]. Soon after the two first bacterial genomes from *Haemophilus influenzae *[2] and *Mycoplasma genitalium *[3] were sequenced, Mushegian and Koonin compared them and proposed 256 genes as a close estimate of a minimal gene set [4]. After this pioneering work, many computational analyses were performed [5-14]. In general, computational analysis is likely to underestimate a minimal gene set, because it considers only orthologous genes. By contrast, for a substantial number of essential functions, non-orthologous, and in some cases non-homologous, genes play the same role in different organisms. The existence of two or more distinct (distantly related or non-homologous) sets of genes that are responsible for the same function in different organisms is called non-orthologous gene displacement (NOGD). Wider genome comparisons have revealed that NOGD even occurs with essential genes, including the central components of the translation, transcription and, especially, replication machineries [1].

In the experimental approach, the essential genes that are indispensable for cell growth are determined by large-scale gene disruption, and they are considered to comprise a minimal gene set. The first experimental attempt along this line was performed by Itaya, before the advent of comparative genomics [15]. He investigated 79 random gene-knockouts in *Bacillus subtilis*, and found that six of them were lethal. Based on this ratio, he estimated the minimal genome size could be 318~562 kbp (270~470 genes, if one protein is 400 aa long). Many subsequent experimental reports utilized individual knockouts [16-18], RNA interference [19], transposon mutagenesis [20-25], antisense RNA [26,27] and high-throughput gene disruption [28]. Because a gene-knockout may just retard cell growth, the numbers of essential genes tend to be overestimated. In contrast, individual gene-knockout studies might underestimate the number of a minimal gene set for a metabolic system, because simultaneous gene knockouts tend to be lethal [12]. In addition, the estimation of essential genes depends on the experimental conditions, such as nutrients contained in culture media.

Considering these difficulties in detecting a minimal gene set by both the computational and experimental approaches, we adopted a different strategy. Instead of a minimal gene set, we computationally explored a minimal cellular-function set. The cellular functions are functional modules composed of a group of genes, for example, glycolysis, TCA cycle and aminoacyl-tRNA biosynthesis. Since one of the final aims of minimal gene set determination is to reveal the functional components of a living cell, and these components are sometimes debated in terms of the combination of the cellular functions, detection of the minimal cellular-function set is a more direct method. In addition, this approach is more robust, because the acceptance of a given cellular function could be possible regardless of NOGD (see Methods). In this work, we regard the KEGG (Kyoto Encyclopedia of Genes and Genomes) pathway maps as the entities of the cellular functions. The KEGG database classifies the genes of sequenced genomes into more than 100 functional modules, named pathway maps, in which the reactions, substrates and products of the corresponding genes (proteins) are shown. Based on this information, not only the network of genes and compounds, but also the network of pathway maps (cellular-functions) can be illustrated.

## Methods

### Outline

**Figure 1:**
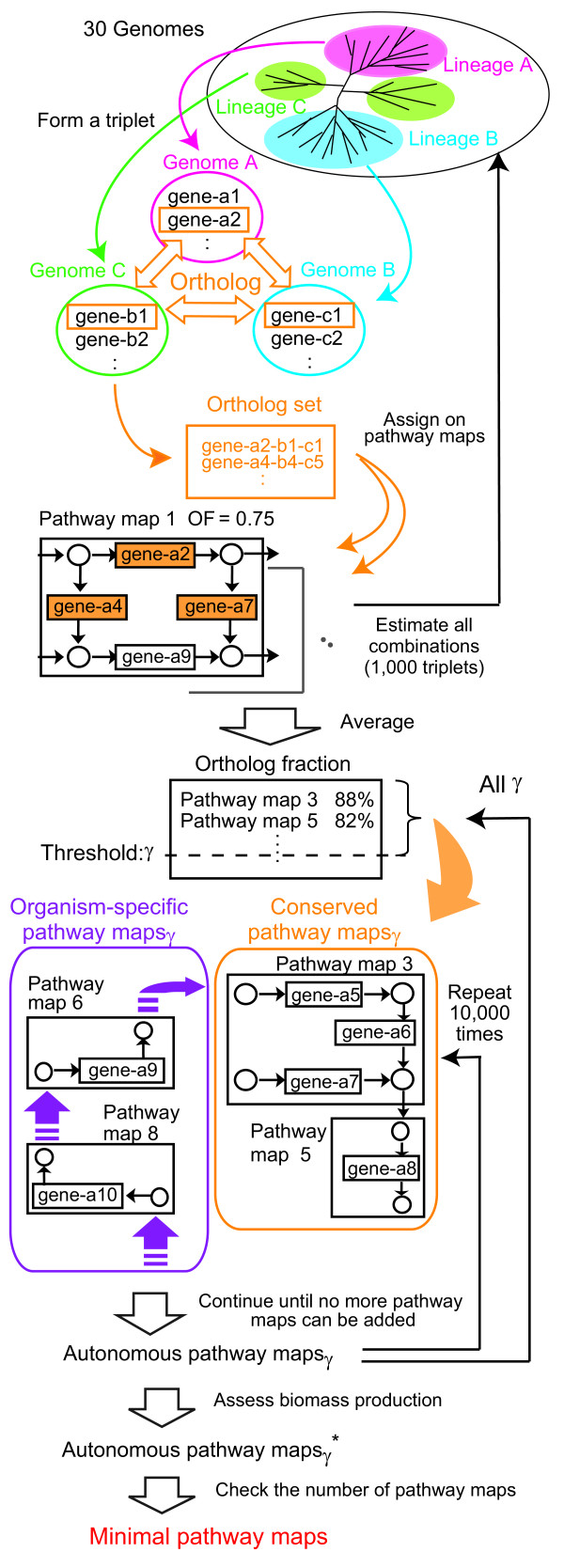
**Schematic procedure to derive minimal pathway maps**.

Figure 1 shows the schematic procedure for determining a pathway map set. It is divided into three parts. In the first part, the conserved pathway maps among many genomes are determined, based on the comparative genomics. In the second part, by checking the compounds imported to and synthesized in the conserved pathway maps, an initial pathway-map network is obtained. Finally, the pathway maps that provide the necessary compounds for the conserved pathway maps are connected to the pathway-map network, until there are no more suitable pathway maps to be added. We regard the final pathway-map network as a candidate for the minimal pathway map (cellular-function) set, and assessed them in terms of biomass production and the number of components (explained later).

### Representative genomes and orthologous genes

As of December 2008, more than 700 bacterial genomes have been completely sequenced. We divided them into three lineages: proteobacteria, firmicutes, and others, with reference to the classical phylogenetic classification (data from KEGG). We selected 10 representative genomes from each lineage, taking the genome size, the phylogenetic distance, and the annotation availability into account (see the Legend of Additional file 1: Table S1 for detail). Thus there are three lineages, each composed of 10 genomes, in the total of 30 genomes (Additional file 1: Table S1). Within a given triplet composed of three genomes, one from each lineage, exhaustive homology searches were performed. Conserved orthologous genes among the three genomes [29] were detected by the bidirectional best-hits method [30]. All of the triplet combinations (1,000) were examined. The influence of the selection of representative genomes is shown in Additional file 1: Table S2.

### Ortholog fraction

The KEGG database provides classifications of functionally identified genes of available genomes and presents them as more than 100 functional modules, "reference pathway maps". For each reference pathway map, the customized pathway map for each genome (organism) was constructed by highlighting the genes assigned to the pathway map. We evaluated the evolutionary conservation of each pathway map, based on the conserved orthologous genes derived as above. For the KEGG pathway maps of a given genome in a triplet, all of the conserved orthologous genes were assigned. Since the total number of genes assigned to a pathway map depends on the genome (organism), we defined the ortholog fraction (OF) of pathway map *i *as,

where *N*_*i*, *orth *_is the number of orthologous genes detected in pathway map *i *using a given triplet, and *Na*_*i*, *min *_is the minimum total-number of genes appearing in pathway map *i *of the three genomes. *OF*_*i *_was averaged over 1,000 triplets. The pathway maps with high OF values are considered to be the "conserved pathway maps", and they will be the "core" of a minimal cellular-function set. Referring to the descending order of OF values, we chose γ pathway maps and classified them as the conserved pathway maps_γ_.

### Reconstruction of the metabolic network in a pathway map

**Figure 2:**
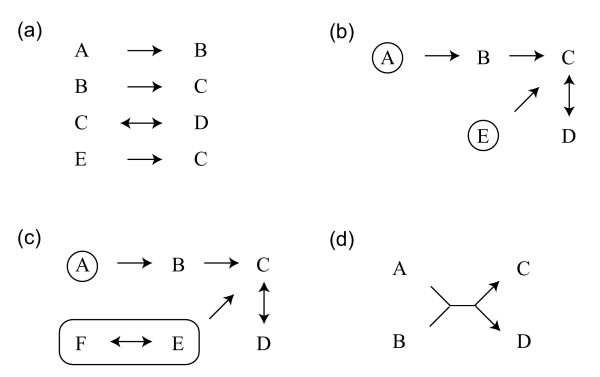
**Reconstruction of the metabolic network from chemical reactions**. (a) Chemical reactions in the binary relation with reversible/irreversible information (arrows). (b) The reconstructed network using chemical reactions in (a). Enclosed characters indicate the initial substrates. (c) The reconstructed network using chemical reactions in (a) and E ↔ F. (d) The reconstructed network from the chemical reactions A + B → C + D. it represents two initial substrates are required to produce each of two products.

A metabolic network within each pathway map was reconstructed, based on the enzymatic reactions of a particular organism provided by the KEGG API service and the reaction_mapformula.lst file on the KEGG FTP site, where the substrates and the products for all reactions in a pathway map are described, mainly as a binary relation (Figure 2a). We used *Escherichia coli *(*E. coli*) and *Bacillus subtilis *(*B. subtilis*) as the model organisms in this work, because there are many experimental and computational data that can be compared. In the network reconstruction process, we regarded the same compounds as one node, and a substrate-product relation as an edge. Therefore a set of reactions in a pathway map was transformed into a unique network (Figure 2b). The reversibility or irreversibility assigned to each reaction in the reaction_mapformula.lst file indicates the stream of compounds in the reconstructed network. We regarded compounds at the upstream termini as the initial substrates (circles in Figure 2b), and all the compounds in the network as products of the pathway map. When a network terminus consists of many initial compounds that are connected by reversible reactions, either one of them can be the initial substrate at the terminus (Figure 2c). We assumed that if all the initial substrates of a pathway map were provided, then all of the reactions would take place there, and all products in the pathway map synthesized. Cofactors were taken into account only if they were described in the chemical reactions as substrates or products. Because such cases are unlikely to be common, cofactors were implicitly assumed to be abundant in the cell, and used if they were required. When a compound is only produced or only consumed in the pathway map, a gap (dead end) exists. At this stage we did not take into account the dead end of the pathway maps. The only-produced compounds are the downstream termini of the network, and the only-consumed compounds are the initial substrates. When the chemical reactions are presented in the indescribable form by the binary relation as, A + B → C + D, both A and B should be the substrates for each of C and D. The network in this case is shown in Figure 2d.

### Construction of the pathway-map network

We randomly selected an initial substrate (in the above case, exceptionally, both A and B) in a pathway map, and tried to connect it to the same compound in another pathway map, regardless of the dead end material. If it was possible, two pathway maps were linked with a directed edge, and a new large network with a new set of initial substrates and products was created. Repeating this procedure for all conserved pathway maps_γ_, an initial pathway-map network was constructed. It should be noted that the initial substrates and network configuration of the initial pathway-map network depended on the order of selection of the initial substrate and the product being connected. In our method, the only-consumed dead end compounds were supplied from the other pathway maps, and the only-produced dead end compounds were assumed to be initial substrates for the other pathway maps or removed by virtual transporters.

### Extension of the pathway-map network

A pathway map that can provide the initial substrates of the pathway-map network was chosen randomly from those other than the components of the pathway-map network, and was connected into the pathway-map network to create a new one. In this process, only a part of the pathway map, i.e., minimal sequential chemical reactions necessary to synthesize the initial substrate, was connected, so that the extra reactions for the initial-pathway map network were excluded. Since the added pathway maps are non-conserved and depend on the organism, we called them the organism-specific pathway maps. Referring to each of the *E. coli *and the *B. subtilis *pathway maps, this selection and connection process was repeated until there were no more pathway maps to be connected. The resultant network defined the network of "autonomous pathway maps", because it was expected to synthesize most of the necessary compounds inside the network. The initial substrates of the autonomous pathway maps were defined as nutrients imported from the extracellular environment. We generated 10,000 autonomous pathway maps from the initial pathway-map construction process using different random seeds. As the autonomous pathway-map network depends on the γ parameter (the number of conserved pathway maps), we started from the conserved pathway maps_γ _and constructed 10,000 patterns of the autonomous pathway maps_γ_, at each γ.

### Estimation of minimal pathway maps

We assumed the autonomous pathway maps have to synthesize at least indispensable compounds for the organism by themselves. The attained autonomous pathway maps were assessed to determine whether they satisfied this condition. As the indispensable compounds, we employed the biomass components estimated by Feist et al. for *E. coli *[31] and by Oh et al. for *B. subtilis *[32], the numbers of which are 61 and 64, respectively. The biomass components are the major and essential constituents that make up the cellular content of organisms. For *E. coli*, they were determined quantitatively using the dry weight composition data for an average *E. coli *B/r cell, which grew exponentially at 37°C under aerobic conditions in a glucose minimal medium [31]. Among the set of autonomous pathway maps, the one that could synthesize all of the biomass components was selected, and denoted as the "autonomous pathway maps*". Minimal pathway maps were decided as the autonomous pathway maps* composed of a minimal number of pathway maps.

## Results and discussion

### Pathway maps with high ortholog fractions

The ortholog fraction was calculated for each pathway map (see Table 1). It revealed that the pathway maps classified as "genetic information processing", i.e., "ribosome", "aminoacyl-tRNA biosynthesis", "RNA polymerase" and "protein export", have high OF values (Table 1. Also refer to the classification columns in Table 2). "DNA polymerase" is also involved in "genetic information processing", but its OF value is lower than the others. This pathway map includes DNA polymerases I-V and DNA polymerase bacteriophage-type. The genes encoding them, except for DNA polymerase III, are not well conserved. The most conserved pathway map is "riboflavin metabolism", which is a member of the "cofactors and vitamins" category. The other members belonging to this class, i.e., "one carbon pool by folate", "pantothenate and CoA biosynthesis" and "porphyrin and chlorophyll metabolism", also have high OF values. This may reflect the importance of these compounds because cofactors and vitamins are involved in many kinds of reactions.

**Table 1:** Pathway maps with more than 50% ortholog fractions.

Rank	Pathway map	OF (%)
1	Riboflavin metabolism	90.8
2	Ribosome	85.4
3	Aminoacyl-tRNA biosynthesis	83.9
4	RNA polymerase	80.8
5	One carbon pool by folate	79.4
6	Peptidoglycan biosynthesis	76.2
7	Pantothenate and CoA biosynthesis	70.8
8	Porphyrin and chlorophyll metabolism	69.4
9	Protein export	67.8
10	Valine, leucine and isoleucine biosynthesis	67.6
11	Phenylalanine, tyrosine and tryptophan biosynthesis	65.8
12	Histidine metabolism	65.8
13	Purine metabolism	65.6
14	Lysine biosynthesis	63.1
15	Pyrimidine metabolism	63.0
16	Aminosugars metabolism	60.9
17	DNA polymerase	59.7
18	Fatty acid biosynthesis	58.9
19	Urea cycle and metabolism of amino groups	57.3
20	Glutamate metabolism	55.2
21	Biosynthesis of steroids	55.0
22	Folate biosynthesis	54.0
23	Alanine and aspartate metabolism	53.5
24	Arginine and proline metabolism	52.3
25	Glycerophospholipid metabolism	52.0
26	Carbon fixation	51.7
27	Pentose phosphate pathway	51.0

### Decision of minimal pathway maps

**Figure 3:**
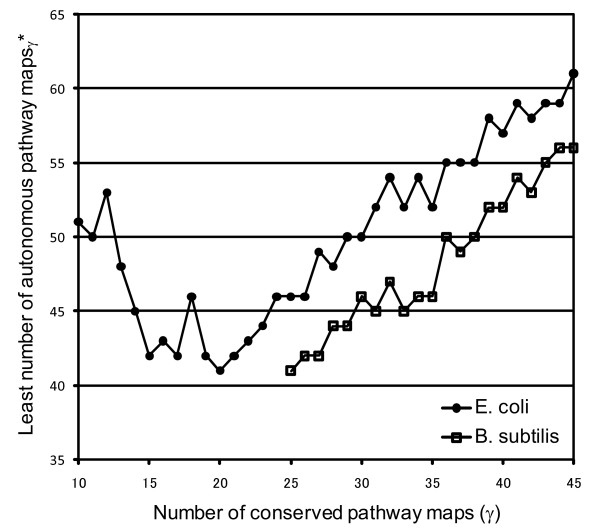
Least number of autonomous pathway maps_γ _* against the number of conserved pathway maps, γ.

We defined γ pathway maps in the descending order of OFs as the conserved pathway maps_γ_. Starting from the conserved pathway maps_γ_, 10,000 sets of organism-specific pathway maps_γ _were determined by expanding the pathway-map networks described in the Methods. The autonomous pathway maps_γ _of *E. coli *and *B. subtilis*, at each γ, consisted of the conserved pathway maps_γ _and the organism-specific pathway maps_γ_. These sets of autonomous pathway maps_γ _were, in turn, used to determine the minimal pathway maps. Initially, the autonomous pathway maps_γ _that could synthesize all the biomass components were selected (i.e., the autonomous pathway maps_γ _*). In Figure 3 the least number of the autonomous pathway maps_γ _* at each γ is plotted against the number of the conserved maps, γ. The least numbers of autonomous pathway maps_γ _* are at a minimum when γ s are 20 (*E. coli*) and 25 (*B. subtilis*). Therefore, minimal pathway maps for *E. coli *and *B. subtilis *were determined from the autonomous pathway maps_20 _* and the autonomous pathway maps_25 _*, respectively, as those composed of the minimal number of the pathway maps.

In Figure 3, the least number of the autonomous pathway maps_γ _* for *B. subtilis *only increases gradually. This indicates that the first time for the autonomous pathway maps to synthesize all the biomass components is at γ = 25. Note that synthesizing all the biomass components is easy if the total number of autonomous pathway maps is large, because the larger the total number is, the greater the products. By contrast, if the initial conserved pathway maps to be extended are not appropriately prepared, the extension process will be terminated before the pathway map network develops. Thus, the conserved pathway maps_25 _of *B. subtilis *provide a good starting point so that the resultant autonomous pathway maps can synthesize all the biomass components. In the case of *E. coli*, the autonomous pathway maps_γ _are able to synthesize all the biomass components at γ = 10, but the least number of the autonomous pathway maps_γ _* decreases by the increment of γ, and reaches a minimum value at γ = 20. After that, it increases as in *B. subtilis*. It also indicates that the conserved pathway maps_20 _of *E. coli *provide a good starting point to be the autonomous pathway maps*, composed of a necessary and sufficient number of the pathway maps.

### Conserved pathway maps

The components of minimal pathway maps for each of the organisms are shown in Table 2. The conserved pathway maps are denoted as "C" (Conserved) in the "MPM" (Minimum Pathway Maps) columns in Table 2. In the left column of the Table, the KEGG classifications of the pathway maps are also indicated. The conserved pathway maps of both organisms contain those classified as "carbohydrate", "lipid", "nucleotide", "amino acid", "glycan", "cofactors and vitamins" and "genetic information processing". They do not, however, include any pathway maps classified as "energy", "other amino acids", "polyketides and nonribosomal peptides", "secondary metabolites", "xenobiotics", "environmental information processing" and "cellular processes".

**Table 2:** Components of minimal pathway maps, experimentally-derived and computationally-derived essential pathway maps for *E. coli *and *B. subtilis*, and components of pathway maps for *B. aphidicola APS*

Classification^1^		Pathway map^2^	*E. coli*					*B. subtilis*			
General	Minor		MPM^3^	%^4^	Exp^5^	Com^6^	Par^7^	MPM^3^	%^4^	Exp^5^	Com^6^
Metabolism	Carbohydrate	Glycolysis/Gluconeogenesis	OS	59.5	X	X	X	OS	85.1	X	X
		Citrate cycle (TCA cycle)	OS	51.4	X	X	X	OS	28.2	X	X
		Pentose phosphate pathway	OS	100.0	X	X	X	OS	100.0	X	X
		Pentose and glucuronate interconversions		62.2					63.4		
		Fructose and mannose metabolism		16.2			X		43.7	X	X
		Galactose metabolism		16.2		X			24.9		
		Ascorbate and aldarate metabolism		21.6					10.1		
		Starch and sucrose metabolism		32.4			X		18.0		X
		Aminosugars metabolism	C		X	X	X	C		X	X
		Nucleotide sugars metabolism							10.3		
		Pyruvate metabolism		64.9	X	X	X		31.2	X	X
		Glyoxylate and dicarboxylate metabolism	OS	91.9			X	OS	92.9		X
		Propanoate metabolism	OS	94.6	X	X	X		92.5	X	X
		C5-Branched dibasic acid metabolism	OS	100.0		X			98.3		
		Butanoate metabolism		35.1			X		30.3		X
	
	Energy	Oxidative phosphorylation				X	X				X
		Carbon fixation	OS	83.8	X	X	X		74.8	X	X
		Reductive carboxylate cycle (CO2 fixation)		56.8		X			45.9		X
		Methane metabolism		86.5			X		67.9		X
		Nitrogen metabolism	OS	97.3				OS	99.9		X
		Sulfur metabolism	OS	100.0			X	OS	99.2		X
	
	Lipid	Fatty acid biosynthesis	C		X	X	X	C		X	X
		Fatty acid elongation in mitochondria		24.3					4.9		
		Fatty acid metabolism	OS	59.5					13.7		
		Synthesis and degradation of ketone bodies							5.2		
		Biosynthesis of steroids		5.4	X	X	X	C		X	
		Glycerolipid metabolism		21.6	X			OS	100.0		
		Glycerophospholipid metabolism	OS	100.0	X	X		C		X	
	
	Nucleotide	Purine metabolism	C		X	X	X	C		X	X
		Pyrimidine metabolism	C		X	X	X	C		X	X
	
	Amino Acid	Urea cycle and metabolism of amino groups	C			X	X	C			
		Glutamate metabolism	C		X	X	X	C		X	X
		Alanine and aspartate metabolism	OS	100.0	X	X	X	C		X	X
		Glycine, serine and threonine metabolism	OS	100.0	X	X	X	OS	99.8	X	X
		Methionine metabolism	OS	97.3	X	X	X	OS	98.3	X	X
		Cysteine metabolism		40.5		X	X		71.8		
		Valine, leucine and isoleucine degradation	OS	100.0			X	OS	99.3		X
		Valine, leucine and isoleucine biosynthesis	C		X	X	X	C		X	X
		Lysine biosynthesis	C		X	X	X	C		X	X
		Lysine degradation		54.1			X		33.2		
		Arginine and proline metabolism	OS	89.2	X	X	X	C		X	
		Histidine metabolism	C			X	X	C			
		Tyrosine metabolism		13.5					13.9		
		Phenylalanine metabolism		67.6					12.5		
		Tryptophan metabolism		24.3			X		21.0		X
		Phenylalanine, tyrosine and tryptophan biosynthesis	C		X	X	X	C		X	X
	
	Other Amino Acids	beta-Alanine metabolism	OS	100.0			X		76.8		
		Taurine and hypotaurine metabolism		5.4			X		25.7		X
		Selenoamino acid metabolism		48.6	X	X	X		32.0	X	X
		Cyanoamino acid metabolism		75.7				OS	96.8		X
		D-Glutamine and D-glutamate metabolism	OS	67.6	X	X	X	OS	57.9	X	
		D-Alanine metabolism		62.2					28.3	X	X
		Glutathione metabolism	OS	100.0			X	OS	60.8		X
	
	Glycan	Lipopolysaccharide biosynthesis		2.7	X		X				
		Peptidoglycan biosynthesis	C		X	X	X	C		X	
		Polyunsaturated fatty acid biosynthesis		70.3					36.3		
	
	Cofactors and Vitamins	Ubiquinone biosynthesis		24.3					6.1	X	
		One carbon pool by folate	C		X	X	X	C		X	X
		Thiamine metabolism		8.1		X	X		29.7	X	
		Riboflavin metabolism	C		X		X	C			
		Vitamin B6 metabolism		27.0			X		22.7		X
		Nicotinate and nicotinamide metabolism	OS	100.0	X		X	OS	100.0	X	
		Pantothenate and CoA biosynthesis	C		X	X	X	C		X	X
		Biotin metabolism	OS	100.0			X	OS	98.1		
		Folate biosynthesis		35.1	X	X	X	C			
		Porphyrin and chlorophyll metabolism	C		X	X	X	C			
	
	Polyketides, Nonribosomal Peptides	Biosynthesis of siderophore group nonribosomal peptides		27.0					6.8		
	
	Secondary Metabolites	Terpenoid biosynthesis			X		X	OS	97.7		
		Alkaloid biosynthesis I		16.2					4.2		
		Alkaloid biosynthesis II		51.4							
		Novobiocin biosynthesis		27.0					4.5		
		Streptomycin biosynthesis		5.4					26.8		
	
	Xenobiotics	Caprolactam degradation		10.8							
		Biphenyl degradation		27.0							
		Toluene and xylene degradation		18.9							
		3-Chloroacrylic acid degradation							3.7		
		Styrene degradation		18.9							
		1,4-Dichlorobenzene degradation		27.0							
		Ethylbenzene degradation		27.0							
		Fluorene degradation		27.0							
		Carbazole degradation		16.2							
		Benzoate degradation via CoA ligation		18.9					15.8		
		Benzoate degradation via hydroxylation		24.3							

Genetic Information Processing	Transcription	RNA polymerase	C		X	X	X	C		X	X
	
	Translation	Aminoacyl-tRNA biosynthesis	C		X	X	X	C		X	X
		Ribosome	C		X	X	X	C		X	X
	
	Folding, Sorting, Degradation	Protein export	C		X	X	X	C		X	X
	
	Replication and Repair	DNA polymerase	C		X	X	X	C		X	X

Environmental Information Processing	Membrane Transport	ABC transporters - General			X	X	X			X	X
	
		Phosphotransferase system (PTS)					X				X
	
	Signal Transduction	Two-component system - General				X	X			X	X

Cellular Processes	Cell Motility	Bacterial chemotaxis - General					X				X
		Flagellar assembly					X				X

^1^KEGG classification (General/Minor) for each pathway map. Minor classifications are denoted as the abbreviations of ''official'' KEGG classifications, e.g, Energy (''Energy metabolism'' in KEGG).

^2^Only the pathway maps referred to in this work are listed.

^3^The components of the minimal pathway maps. C: the conserved pathway maps, OS: the organism-specific pathway maps.

^4^The percentage of appearance of organism-specific maps in the 54 autonomous pathway maps_20_* of *E. coli *(or 3,391 autonomous pathway maps_25_* of *B. subtilis*). Only the pathway maps appeared at least once are shown.

^5^The experimentally-derived essential pathway maps.

^6^The computationally-derived essential pathway maps.

^7^The pathway maps for *Buchnera aphidicola APS*.

### Organism-specific pathway maps

The organism-specific pathway maps in the minimal pathway maps are denoted as "OS" (Organism Specific) in the "MPM" columns in Table 2. In the "carbohydrate" category, "glycolysis/gluconeogenesis", "citrate cycle (TCA cycle)" and "pentose phosphate pathway" were selected. It is also notable that in the "amino acid" category, the components of minimal pathway maps are identical in *E. coli *and *B. subtilis*, whereby in *B. subtilis*, "alanine and aspartate metabolism" and "arginine and proline metabolism" were identified as the conserved pathway maps, whereas they were chosen as the organism-specific pathway maps in *E. coli*. This indicates that even though their degrees of conservation are marginal, they are necessary to constitute the minimal pathway maps. In the "other amino acids" category, β-amino acid (only in *E. coli*) and D-amino acid metabolisms were required for the minimal pathway maps. In addition, the pathway maps in the "energy", "lipid", "cofactors and vitamins" and "secondary metabolites" (only in *B. subtilis*) categories were also selected. In the "carbohydrate" and "energy" categories, all of the pathway maps other than "aminosugars metabolism" were selected as the organism-specific pathway map, not as the conserved pathway map. This result means that catabolism is essential for cellular life, but is not conserved throughout the bacterial genomes, probably because bacteria have evolved to adapt to a variety of environments. During the evolutionary process, bacteria that succeeded in obtaining or adjusting genes to effectively utilize the nutrients around them could survive. Consequently, their catabolic pathway maps have diverged.

As described in the Methods, 10,000 sets of autonomous pathway maps of *E. coli *and *B. subtilis *for each were determined for every γ, to decide the minimal pathway maps. The autonomous pathway maps thus obtained show various combinations of the pathway maps, reflecting the variety of the organism-specific pathway maps selected. Only a part of them, named "autonomous pathway maps*", can synthesize all biomass components required to be an autonomous cell. To characterize the organism-specific pathway maps included in the minimal pathway maps, we calculated the percentage appearance of each pathway map in about 54 sets of autonomous pathway maps_20 _* in *E. coli *and 3,391 sets of autonomous pathway maps_25 _* in *B. subtilis *("%" columns in Table 2). The discrepancy in the total number of the autonomous pathway maps* for each organism is due to the difference in the total number of conserved pathway maps. It is difficult to obtain autonomous pathway maps* using a small number of conserved pathway maps. Most organism-specific pathway maps in the minimal pathway maps are adopted almost always in the autonomous pathway maps*. For example, the organism-specific pathway maps in the "amino acid" category, all appear with more than, or nearly equal to, 90%. By contrast, some examples show the participation in the minimal pathway maps and the percentage of appearance in the autonomous pathway maps* are not necessarily mutually related: some participants in the minimal pathway maps, e.g., "citrate cycle (TCA cycle)", are not frequently accepted in the autonomous pathway maps*, whereas the other pathway maps, e.g., "methane metabolism" in *E. coli*, are accepted with a very high ratio, but do not participate in the minimal pathway maps. This is partly explained by the number of products and initial substrates for the pathway map. When a pathway map includes several products that can be the initial substrates of the pathway-map network, this pathway map will be frequently accepted at the "extension of the pathway-map network" process. However, if the newly connected pathway map requires many kinds of initial substrates, the extended pathway-map network needs many additional pathway maps that will supply the initial substrates. Consequently, the resultant autonomous pathway maps* cannot be minimal pathway maps. We found that the "reductive carboxylate cycle (CO2 fixation)" is almost equivalent (reverse reaction) to the "citrate cycle (TCA cycle)" from the viewpoint of logistics of compounds, and they were alternatively selected in many cases (the sum of their percentages of appearance is around 100%). But, there are dead ends in "reductive carboxylate cycle (CO2 fixation)" (at least ATP citrate synthase and 2-oxoglutarate synthase are missing in *E. coli*). Subsequently, this pathway map requires more initial substrates than the "citrate cycle (TCA cycle)". Furthermore, we noticed that 64 pathway maps (excluding the conserved pathway maps) appeared at least once for the autonomous pathway maps_20 _* in *E. coli *(Table 2). Considering that the number of the organism-specific pathway maps in the minimal pathway maps is 21, only one third (21/64) can be the components of the minimal pathway maps. This ratio is almost the same for *B. subtilis *(16/52). These results indicate that there are many possibilities to synthesize the biomass components from extracellular nutrients, i.e., many solutions to realize the autonomous pathway maps*, if the total number of the pathway maps is unlimited.

### Network of minimal pathway maps

**Figure 4:**
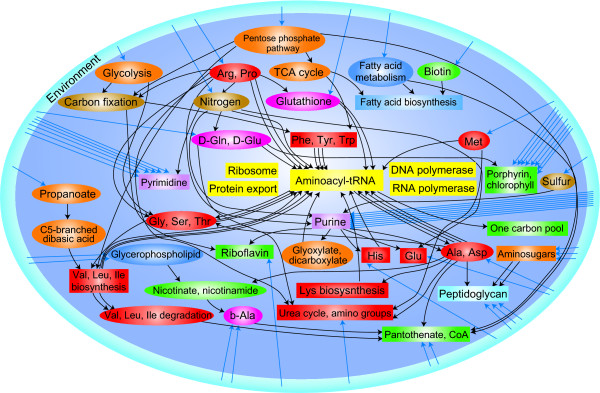
**Network of minimal pathway maps for *E. coli***. The box and ellipse nodes represent the conserved pathway maps_20 _and the organism-specific pathway maps_20_, respectively. The colors indicate the category of the pathway maps in the KEGG database (see Table 2). Orange, "carbohydrate"; brown, "energy"; blue, "lipid"; purple, "nucleotide"; red, "amino acid"; magenta, "other amino acids"; sky-blue, "glycan"; green, "cofactors and vitamins" and yellow, "genetic information processing". The notes in the boxes are abbreviations for the pathway maps, using a few words from the long descriptions in Table 2. Note that the amino acids (colored red and magenta) are denoted by three letter codes. A directed edge indicates the flow of one compound. The blue directed edges entering the central cell from the environment indicate the imports of nutrients. The exchanged compounds between pathway maps and the nutrients imported from extracellular environment are listed in Additional file 2: Table S3.

The network of minimal pathway maps of *E. coli *is shown in Figure 4. The compounds exchanged between pathway maps, and nutrients imported from the extracellular environment are listed in Additional file 2: Table S3. The network starts from pathway maps mostly in the "carbohydrate" (orange nodes) and "energy" (brown nodes) categories that function in catabolism. "Glycolysis/gluconeogenesis", "pentose phosphate pathway" and "propanoate metabolism" catabolize imported nutrients into products and provide them as the initial substrates for "citrate cycle (TCA cycle)", "carbon fixation" and "C5-Branched dibasic acid metabolism", and further catabolism occurs there. Subsequently, the products are used for anabolism. Most pathway maps of the "amino acid" category (red nodes) utilize products of pathway maps in the "carbohydrate" or "energy" category, but a few of them utilize products of pathway maps in the "nucleotide" (purple nodes), "other amino acids" (magenta nodes) or "genetic information processing" (yellow nodes) category. Around one of the termini of the network, "aminoacyl-tRNA biosynthesis" (yellow rectangle) exists. Most of the amino acids fed into this pathway map are synthesized by the pathway maps in the "amino acid" category. However, glycine and cysteine are provided from "glutathione metabolism", glutamine is from "nitrogen metabolism" and glutamate is from "D-glutamine and D-glutamate metabolism". This is due to the overlaps of pathway maps, that is, some of the amino acids synthetic reactions occur in pathway maps that are not classified as "amino acid". Note that the number of substrates into "aminoacyl-tRNA biosynthesis" is 21. This is because "aminoacyl-tRNA biosynthesis" synthesizes not only 20 aminoacyl-tRNAs, but also N-formylmethionyl-tRNA using 10-formyl-THF. Most pathway maps belonging to the "lipid" (blue nodes) or "cofactors and vitamins" (green nodes) category are closely connected in the network. All the pathway maps are connected into one network, except four pathway maps belonging to the "genetic information processing" category. Because any reactions are not described in the reaction_mapformula.lst file for them, they cannot have any links to the other pathway maps via the substrates and products. This overall compound-flow looks reasonable for an ideal metabolism of a living cell.

### Comparison with experimental data

We compared minimal pathway maps of *E. coli *with the data on the "Profiling of *E. coli *Chromosome" (PEC) database that presents 302 essential genes [33]. Also minimal pathway maps of *B. subtilis *were compared with the result of a systematic gene knockout experiment of *B. subtilis *that detected 271 essential genes [16]. We defined the "experimentally-derived essential pathway maps" as those including at least two essential genes. The threshold "two" was selected, because using this value, we could define a comparable number of "experimentally-derived essential pathway maps" to compare them with the minimal pathway maps. It should be noted that approximately half of the genes coded in the *E. coli *genome are multiply assigned to more than or equal to two pathway maps. Therefore, the pathway map that includes at least one essential gene does not necessarily represent the experimentally-derived essential pathway map. We also employed the definition by which we could achieve the highest correlation between our results and the experimental results. If both results were different, there would be no mutual relation, even though we employed a biased threshold. We discuss other definitions in the "Remarkable features of network construction" section.

Among 114 pathway maps presented in the KEGG database, 38 were determined to be experimentally essential for *E. coli*. Among those, 30 are in common with minimal pathway maps in *E. coli*. Also 37 were determined to be experimentally essential for *B. subtilis*. They include 27 pathway maps that are in common with the minimal pathway maps of *B. subtilis *(see the pathway maps marked by "C" or "OS" in the "MPM" columns and "X" in the "Exp" columns in Table 2). We calculated the Jaccard coefficients between our results and experimental results (the number of pathway maps in both results/the number of pathway maps appeared in either or both) for every minor classification of metabolism in Table 2, and summarized them in Table 3. In *E. coli*, the "carbohydrate", "nucleotide", "amino acid", "glycan" and "cofactors and vitamins" categories are very consistent (the Jaccard coefficients ≥ 0.5), but "energy", "lipid", "other amino acids" and "secondary metabolites" are less consistent (< 0.5). The Jaccard coefficients for whole pathway maps (all categories) are 0.61 for *E. coli *and 0.53 for *B. subtilis *("All" row in Table 3). Some of the discrepancies are due to the experimental environment. The inactivation experiments were carried out with a rich medium. When we derived minimal pathway maps, however, the initial substrates were the materials that cannot be synthesized in any pathway map. Catabolism ("carbohydrate" and "energy" categories) and "cofactors and vitamins" categories are considered to be strongly affected by nutrient composition. In the experiment, some cofactors, e.g., folate and pho-CoA, might be transported from the medium into the cell [16] and thus they do not need to be synthesized, while we required minimal pathway maps to synthesize as many requisite compounds as possible. The very high consistencies seen in the "amino acid" and "nucleotide" categories might show that in the experiments, amino acids and nucleotides were provided in the LB medium, but not abundantly [16].

**Table 3:** Jaccard coefficients between our results and experimental, computational, or parasitic results in each minor classification and in whole pathway maps.

Classification	Experiment		Computation		Parasite
	*E. coli*	*B. subtilis*	*E. coli*	*B. subtilis*	*B. aphidicola*
Carbohydrate	0.63	0.50	0.67	0.50	0.55
Energy	0.33	0.00	0.20	0.33	0.40
Lipid	0.40	0.75	0.50	0.25	0.25
Nucleotide	1.00	1.00	1.00	1.00	1.00
Amino Acid	0.73	0.73	0.83	0.67	0.79
Other Amino Acids	0.25	0.00	0.38	0.33	0.60
Glycan	0.50	1.00	1.00	0.00	0.50
Cofactors and Vitamins	0.71	0.33	0.38	0.25	0.67
Secondary Metabolites	0.00	0.00	0.00	0.00	0.00

All	0.61	0.53	0.60	0.45	0.59

### Comparison with computational data

The results of computational studies, the persistent genes of *E. coli *and the functional genomic core of *B. subtilis*, were compared with our results. The former results are the orthologous genes conserved in most of 228 bacterial genomes [14] and the latter results are the genes adopting highly biased codon usage[11]. These two data sets were selected from many studies on the minimal gene sets, because they are based on *E. coli *and *B. subtilis *genomes, anonymously accessible, and easy to convert from original ID to the KEGG ID. The "computationally-derived essential pathway maps" were assigned by the same process described in the "comparison with experimental data" section, but we used thresholds 3 and 1 for *E. coli *and *B. subtilis*, respectively, because these values were appropriate to identify a comparable number of computationally-derived essential pathway maps, so that we compared them with the minimal pathway maps.

42 and 46 computationally-derived essential pathway maps were defined for *E. coli *and *B. subtilis*, respectively. Among them, 31 and 27 were in common with the minimal pathway maps of *E. coli *and *B. subtilis*, respectively. The good consistency observed in *E. coli *(the Jaccard coefficient, 0.60 in total. See Table 3) could be due to the similarity of methodologies that detect minimal pathway maps and persistent genes. Both methods relied on the conservation of genes among many bacterial genomes. The lower consistency in *B. subtilis *(0.45) might be explained as follows. We noticed that the computationally-derived essential pathway maps for *B. subtilis *were abundant in catabolism ("carbohydrate" and "energy" categories). Such pathway maps occupy 35% of all computationally-derived essential pathway maps. On the other hand, the occupancies of pathway maps of catabolism in our results and in the experimental results, are only 17% and 22%, respectively. This is probably because the genes used in catabolism are likely to be under codon usage, to be expressed abundantly and ubiquitously, so that the bacteria adapt a specific environment in which they should survive.

### Comparison with parasitic genomes

*Buchnera aphidicola *(*B. aphidicola*) strains (*APS*, *Sg*, *Bp*, *Cc*, *5A *and *Tuc7*) and *Wigglesworthia glossinidia *(*W. glossinidia*) are parasitic organisms and phylogenetically close to *E. coli *[34]. We compared the minimal pathway maps of *E. coli *with their genomes. The pathway maps that the genomes hold were taken from the KEGG database. In Table 2, the pathway maps of *B. aphidicola APS *are shown in the "Par" (Parasite) column. Against these data, the Jaccard coefficients of minimal pathway maps of *E. coli *are calculated and shown in the "Parasite" column in Table 3.

In the "carbohydrate" and "lipid" categories, the coefficients were lower than those against the experimental and the computational results, but in the "energy" and "other amino acids" categories, the coefficients were higher than those against the experimental and the computational results. The total Jaccard coefficients against the data of *B. aphidicola APS*, *Sg*, *Bp*, *Cc*, *5A*, *Tuc7 *and *W. glossinidia*, were 0.59, 0.59, 0.59, 0.56, 0.59, 0.60 and 0.52, respectively, indicating minimal pathway maps show better consistency with the data of *B. aphidicola *strains than with the data of *W. glossinidia*. The minimal pathway maps were also compared with the KEGG pathway maps of *Mycoplasma genitalium*. This organism is not a close relative of *E. coli*. The Jaccard coefficient was 0.49 in total.

The high Jaccard coefficients between the minimal pathway maps of *E. coli *and the pathway maps of several parasitic genomes imply that the minimal cellular functions are represented in the minimal pathway maps of *E. coli*. Also the slight differences seen in the Jaccard coefficients may reflect the phylogenetic distances between *E. coli *and the parasites.

### Remarkable features of network construction

We demonstrated that there were good consistencies in the comparisons between the minimal pathway maps and the experimental, computational and parasitic data. However, we noticed that these results depended on how the essential genes to the essential pathway maps were converted. We used "number" of essential genes in the pathway maps to define the essential pathway maps. When we employed the "fraction" of essential genes in the pathway maps, as was applied to define the conserved pathway maps (OF value), instead of the number, the results were slightly different. In both experimental and computational results for *E. coli*, the fractions of essential genes for all pathway maps in "genetic information processing" were higher than 30% (data not shown). However, the fractions of essential genes for all pathway maps in catabolism ("carbohydrate" and "energy" categories) were lower than 30%, except the fraction for "C5-Branched dibasic acid metabolism" in the computational results (50%). This is because the computationally-determined essential genes are the conserved genes, and genes in "genetic information processing" are strongly conserved. Also these genes tend to code proteins that have no substitutions, e.g., each of the ribosomal proteins. Disrupting them is likely to be lethal. Apparently, the computational method referring only to the gene conservation can hardly clarify the significance of catabolism. On the other hand, we first identify the conserved pathway maps that include conserved orthologous genes. Subsequently, the pathway maps that supply the substrates for the conserved pathway maps are identified. In this case, the pathway maps in catabolism are naturally introduced. Although this method relies on the reliability of chemical reactions or pathway-map network data employed, the framework is very simple. The significance of catabolism may be still under discussion and one may consider only the genes in "genetic information processing" and a small number of additional genes are enough to constitute a living cell in very rich media, even though there are no genes in catabolism. We cannot argue for the possibility of such a virtual organism at this stage, but we can point out that in each bacterial genome the portion of genes for catabolism is considerable (in *E. coli *7%), and to shed light on their significance, our method is effective. We consider this methodology provides an alternative way to explore a minimal cellular-function set, other than current experimental and computational approaches.

## Conclusion

The method to evaluate the minimal KEGG pathway maps proposed here identified 41 pathway maps, including 20 conserved pathway maps and 21 organism-specific pathway maps for *E. coli*, and 41 pathway maps, including 25 conserved pathway maps and 16 organism-specific pathway maps for *B. subtilis*. The conserved pathway maps include many pathway maps classified as "genetic information processing", whereas the organism-specific pathway maps mainly include pathway maps for catabolism, reflecting evolutionary aspects. The consistencies between minimal pathway maps and the experimental, computational, and parasitic data indicate that our procedure is realistic.

In the case of KEGG data analysis, since our method to detect organism-specific pathway maps is applicable to only the enzymatic reactions, it is insufficient to estimate the essentialities of "membrane transport", "signal transduction" or "cell motility", for which the chemical reactions are not provided in the reaction_mapformula.lst file. However, our method can be applied to the other data for biochemical reactions, instead of the KEGG data, e.g., a genome-scale metabolic model of *E. coli*, iAF1260 [31] and that of *B. subtilis*, iYO844 [32]. By analyzing the other data as well as modifying our algorithm, we can refine our results.

As mentioned in the Background, a minimal genome and a minimal cellular-function set depend on the environment or nutrients, and their general definitions are very difficult. However, an estimation of minimal cellular-functions to realize a specific biological system is useful to design an efficient biological process, from the viewpoints of synthetic biology and cell engineering. For instance, we could design a bacterial genome that will degrade harmful chemicals, such as dioxin, or synthesize beneficial materials, such as ethanol, in large quantities through photosynthesis, by considering only minimal pathway maps related to their efficient catalysis.

## Authors' contributions

YA performed the computational experiments, analyzed the data and wrote the manuscript. MO supervised the study, analyzed the data and wrote the manuscript.

## Supplementary Material

Additional file 1 **Table S1 and S2.**. The 30 representative genomes (Table S1) and the pathway maps with high OF values recalculated using 30 genomes that were partly different from the original 30 genomes (Table S2). Click here for file

Additional file 2 **Table S3**. Compounds exchanged between the pathway maps and nutrients imported from extracellular environment in Figure 4. Click here for file
