# Supplementary material for: An evaluation of minimal cellular functions to sustain a bacterial cell
Source: BMC Syst Biol. 2009 Nov 28;3:111. doi: 10.1186/1752-0509-3-111 (PMC2789071; doi:10.1186/1752-0509-3-111)
Supplement: Additional file 1 — Table S1 and S2.. The 30 representative genomes (Table S1) and the pathway maps with high OF values recalculated using 30 genomes that were partly different from the original 30 genomes (Table S2). [file 1752-0509-3-111-S1.doc]

**Supplementary Material**

**Table S1 : 30 representative genomes**.

| Lineage | Family | Genus | Genus / Species | Size (Mbp) | Gram strain | Oxygen |
| --- | --- | --- | --- | --- | --- | --- |
| Proteobacteria | Proteobacteria | Alphaproteobacteria | Agrobacterium tumefaciens str. C58 | 5.67 | - | Aerobic |
|  |  |  | Gluconobacter oxydans 621H | 2.92 | - | Aerobic |
|  |  |  | Caulobacter crescentus CB15 | 4.02 | - | Aerobic |
|  |  | Betaproteobacteria | Chromobacterium violaceum ATCC 12472 | 4.75 | - | Facultative |
|  |  |  | Bordetella pertussis Tohama I | 4.09 | - | Aerobic |
|  |  | Deltaproteobacteria | Bdellovibrio bacteriovorus HD100 | 3.78 | - | Aerobic |
|  |  |  | Geobacter sulfurreducens PCA | 3.81 | - | Anaerobic |
|  |  | Gammaproteobacteria | Photorhabdus luminescens subsp. laumondii TTO1 | 5.69 | - | Anaerobic |
|  |  |  | Escherichia coli K12 | 4.64 | - | Facultative |
|  |  |  | Xylella fastidiosa Temecula1 | 2.52 | unknown | Aerobic |
| Firmicutes | Firmicutes | Bacillales | Bacillus subtilis 168 | 4.21 | + | Facultative |
|  |  |  | Geobacillus kaustophilus HTA426 | 3.59 | + | Aerobic |
|  |  |  | Listeria monocytogenes EGD-e | 2.94 | + | Facultative |
|  |  |  | Oceanobacillus iheyensis HTE831 | 3.63 | + | Aerobic |
|  |  |  | Staphylococcus aureus MSSA476 | 2.82 | + | Facultative |
|  |  | Clostridia | Clostridium acetobutylicum ATCC 824 | 4.13 | + | Anaerobic |
|  |  |  | Thermoanaerobacter tengcongensis MB4 | 2.69 | - | Anaerobic |
|  |  | Lactobacillales | Enterococcus faecalis V583 | 3.36 | + | Facultative |
|  |  |  | Lactobacillus plantarum WCFS1 | 3.35 | + | Facultative |
|  |  |  | Lactococcus lactis subsp. lactis IL1403 | 2.43 | + | Facultative |
| Others | Actinobacteria | Actinobacteridae | Corynebacterium diphtheriae gravis NCTC13129 | 2.49 | + | Aerobic |
|  |  |  | Leifsonia xyli subsp. xyli str. CTCB07 | 2.58 | - | Aerobic |
|  |  |  | Mycobacterium bovis AF2122/97 | 4.35 | + | Aerobic |
|  |  |  | Nocardia farcinica IFM 10152 | 6.29 | + | Aerobic |
|  |  |  | Symbiobacterium thermophilum IAM 14863 | 3.57 | + | Microaerophilic |
|  | Cyanobacteria | Chroococcales | Synechocystis sp. PCC 6803 | 3.57 | unknown | unknown |
|  |  |  | Thermosynechococcus elongatus BP-1 | 2.59 | unknown | unknown |
|  |  | Gloeobacteria | Gloeobacter violaceus PCC 7421 | 4.66 | unknown | unknown |
|  | Deinococcus-Thermus | Deinococci | Deinococcus radiodurans R1 | 3.28 | + | Aerobic |
|  | Planctomycetes | Planctomycetacia | Rhodopirellula baltica SH 1 | 7.15 | - | Aerobic |

From the completely sequenced bacterial genomes, we removed genomes whose sizes were equal to or smaller than 2.41 Mbp that was the genome size of the largest parasitic bacterium (*Candidatus Protochlamydia amoebophila*) [1] among those we confirmed. The genome with the highest fraction of annotated genes (the lowest fraction of unannotated genes i.e., hypothetical or putative genes) was selected from each genus to remove phylogenetic bias. We divided genomes into three lineages according to the classical phylogenetic classification, so as to make the population of each lineage approximately equal. In each lineage, 10 genomes with higher fractions of annotated genes were selected as the representatives.

**Table S2 : Pathway maps with high OF values recalculated using 30 genomes that were partly different from the original 30 genomes.**

| Rank | |  | | Pathway map | | OF (%) | | Rank in Table1 | |
| --- | --- | --- | --- | --- | --- | --- | --- | --- | --- |
| 1 |  | | Riboflavin metabolism | | 85.6 | | 1 | |  |
| 2 |  | | Ribosome | | 75.8 | | 2 | |  |
| 3 |  | | One carbon pool by folate | | 73.5 | | 5 | |  |
| 4 |  | | Aminoacyl-tRNA biosynthesis | | 71.5 | | 3 | |  |
| 5 |  | | RNA polymerase | | 71.0 | | 4 | |  |
| 6 |  | | Nicotinate and nicotinamide metabolism | | 66.8 | | 33 | |  |
| 7 |  | | Peptidoglycan biosynthesis | | 66.0 | | 6 | |  |
| 8 |  | | Pantothenate and CoA biosynthesis | | 63.6 | | 7 | |  |
| 9 |  | | Histidine metabolism | | 62.3 | | 12 | |  |
| 10 |  | | Aminosugars metabolism | | 60.2 | | 16 | |  |
| 11 |  | | Purine metabolism | | 59.5 | | 13 | |  |
| 12 |  | | Porphyrin and chlorophyll metabolism | | 59.5 | | 8 | |  |
| 13 |  | | Pyrimidine metabolism | | 59.4 | | 15 | |  |
| 14 |  | | Lysine biosynthesis | | 59.1 | | 14 | |  |
| 15 |  | | Protein export | | 58.2 | | 9 | |  |
| 16 |  | | Valine, leucine and isoleucine biosynthesis | | 58.1 | | 10 | |  |
| 17 |  | | Phenylalanine, tyrosine and tryptophan biosynthesis | | 57.1 | | 11 | |  |
| 18 |  | | Urea cycle and metabolism of amino groups | | 55.4 | | 19 | |  |
| 19 |  | | DNA polymerase | | 55.2 | | 17 | |  |
| 20 |  | | Arginine and proline metabolism | | 54.7 | | 24 | |  |
| 21 |  | | Carbon fixation | | 51.7 | | 26 | |  |
| 22 |  | | Glutamate metabolism | | 50.7 | | 20 | |  |
| 23 |  | | Folate biosynthesis | | 50.0 | | 22 | |  |
| 24 |  | | Alanine and aspartate metabolism | | 49.9 | | 23 | |  |
| 25 |  | | Glycerophospholipid metabolism | | 49.4 | | 25 | |  |
| 26 |  | | Biosynthesis of steroids | | 49.3 | | 21 | |  |
| 27 |  | | Cysteine metabolism | | 46.7 | | 29 | |  |

The ortholog fractions (OFs) were calculated using 30 genomes, where five genomes in the “other” category ("Synechocystis sp. PCC 6803", "Thermosynechococcus elongatus BP-1", "Gloeobacter violaceus PCC 7421", "Deinococcus radiodurans R1" and "Rhodopirellula baltica SH 1") were replaced by five genomes of “actinobacteria” ("Clavibacter michiganensis subsp. michiganensis", "Frankia sp. CcI3", "Kineococcus radiotolerans", "Salinispora tropica" and "Thermobifida fusca"). Thus, lineages are composed of three unique families, i.e., proteobacteria, firmicutes and actinobacteria. The ranks of pathway maps with high OF values, calculated using original 30 genomes (Table 1), are also shown. The Spearman's rank correlation coefficient was 0.86 between the original and new results.

**References**

1. Horn M, Collingro A, Schmitz-Esser S, et al.**: Illuminating the Evolutionary History of Chlamydi**ae*. Scien*ce 2004**, 3**04:728-730.
